# The relationship between arterial stiffness index and coronary heart disease and its severity

**DOI:** 10.1186/s12872-021-02350-6

**Published:** 2021-11-08

**Authors:** Longjian Gao, Dasheng Lu, Guangwei Xia, Hao Zhang

**Affiliations:** 1 Department of Cardiology, The Affiliated Hospital of Yangzhou University, 41# Taizhou Road, Yangzhou, 225000 Jiangsu Province China; 2 Department of Cardiology, Hebei Medical University, 361# Zhongshan east road, Shijiazhuang, 050017 Hebei Province China; 3 Department of Cardiology, The Second Affiliated Hospital of Wannan Medical College, 10# Kangfu road, Wuhu, 241000 Anhui Province China

**Keywords:** Arterial stiffness index, Coronary heart disease, Arterial elasticity, Coronary atherosclerosis

## Abstract

**Background:**

Arterial stiffness index (ASI) is closely related to coronary atherosclerosis. This study aims to explore whether ASI can predict coronary heart disease (CHD) and its severity.

**Methods:**

In this study, a total of 726 patients with suspected CHD were recruited. Based on coronary angiography results, the subjects were assigned into three groups: the control group (without obvious coronary artery disease), single-vessel disease group, and multi-vessel disease group (the number of vessels diseased ≥ 2). At the same time, according to the results of angiography, myocardial enzyme spectrum, electrocardiogram, color Doppler echocardiography and clinical manifestations, these patients were divided into four groups: the control group, stable angina (SA) Group, unstable angina (UA) group, and acute myocardial infarction (AMI) group. We have compared whether there were differences in ASI and related baseline data between groups. Receiver operating curve (ROC) analysis was conducted to determine whether ASI could predict CHD and evaluate the severity.

**Results:**

ASI was positively correlated with the number of diseased branches of coronary artery. The value of ASI was increased as the number of the diseased branches increased. The ASI value in the SA group was significantly higher compared with the control group. Furthermore, the ASI value in the UA and AMI groups was remarkably increased compared with the control and SA groups. The results of ROC analysis indicated that the sensitivity and specificity of ASI was 71.0% and 85.4% in diagnosing CHD, respectively. While ASI was used in predicting the severity of CHD, the sensitivity was 72.1% and specificity 57.9%.

**Conclusion:**

ASI is of great value in the diagnosis of coronary heart disease and the prediction of its severity.

## Background

Cardiovascular disease, especially CHD, is one of the important diseases affecting life and health. Although accumulating research has investigated the disease in the past, numerous related issues on diagnosis, treatment and prognosis remained to be resolved [1]. CHD is the synergy of multiple factors. In the past, the research mainly focused on the luminal lesions of coronary artery, especially lipid content, plaque stability and the biological characteristics of the fibrous cap. Recently, since arterial compliance is non-invasive and can be detected before the occurrence of cardiovascular disease, arterial elasticity changes have gained increasing attention.

Arteriosclerosis is an independent risk factor of CHD and other cardiovascular diseases [2, 3]. In recent years, it was believed that the occurrence and development of cardio-cerebrovascular diseases is resulted from the change of arterial wall elasticity (compliance), which predates the structural changes. Thus, research on arterial compliance at an early stage is of great necessity [4]. Arterial compliance mainly reflects arterial diastolic function, it mainly depends on the diameter, stiffness and expandability of blood vessels, which indicates vascular endothelial function to some extent [5, 6]. Therefore, the research on vascular compliance can provide a new target for the treatment of cardiovascular diseases [7]. ASI, an index of vascular elasticity measured by oscillography, can reflect the condition of the entire cardiovascular system. Common indicators of vascular elasticity include pulse pressure, pulse wave velocity (PWV), etc. Studies have shown that ASI was closely related with PWV. Reduced cardiovascular compliance is related with remarkably increased ASI, especially in patients with vascular sclerosis [8].

Based on previous studies, detecting ASI could predict CHD and evaluate its severity. This can provide new reference for the diagnosis and treatment of CHD in the future.

## Methods

### Patients and the groups

**Fig. 1:**
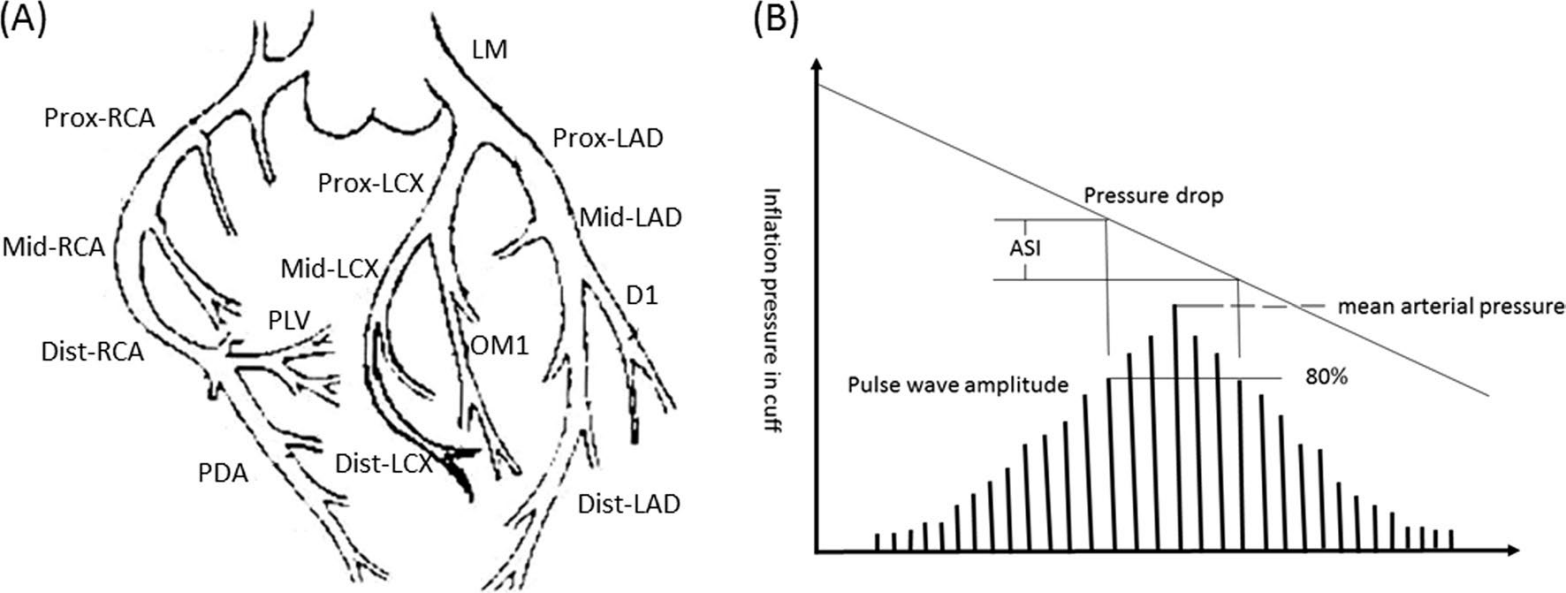
Brief anatomy of coronary artery and basic schematic diagram of ASI detection. **a** Brief anatomy of coronary artery; **b** Basic schematic diagram of ASI detection. Denote: Prox-RCA = Proximal right coronary artery; Mid-RCA = Middle right coronary artery; Dist-RCA = Distal right coronary artery; PDA = Posterior descending artery; PLV = Posterior left ventricular; LM = Left main stem; Prox-LCX = Proximal left circumflex; Mid-LCX = Middle left circumflex; Dist-LCX = Distal left circumflex; OM1 = First obtuse marginal; Prox-LAD = Proximal left anterior descending; Mid-LAD = Middle left anterior descending; Dist- LAD = Distal left anterior descending; D1 = First diagonal

We selected 933 patients with suspected CHD who were hospitalized in the Department of Cardiology, Affiliated Hospital of Yangzhou University from January 2019 to December 2020, finally, 726 patients were selected. Among these patients, patients with AMI mainly come from emergency department, and other patients mainly come from cardiology outpatient department. The exclusion criteria are as follows: 1. Severe liver, kidney and pancreatic diseases; 2. Chronic wasting diseases and malignant tumors; 3. Frequent ectopic arrhythmias; 4. Those who do not cooperate with the inspection. All the subjects underwent coronary angiography based on the requirements of the American Heart College (ACC)/American Heart Association (AHA) coronary angiography guidelines. We mainly observed the lesions of left anterior descending (LAD) branch, left circumflex (LCX) branch, right coronary artery (RCA) and their main branches [9], as shown in Fig. 1a. The severity of coronary artery disease was evaluated according to the results of the examination [10]. According to the number of diseased branches of coronary artery (degree of stenosis ≥ 50%), all the subjects were assigned into three groups, including the control group (no vascular with stenosis degree ≥ 50%), and the single-vessel disease group (one vessel with stenosis degree ≥ 50%), multi-vessel disease group (two or more blood vessels with stenosis degree ≥ 50%). In addition, according to the results of color Doppler echocardiography, electrocardiogram, myocardial enzyme spectrum, and patient's clinical manifestations, the subjects were assigned into four groups, including the control group (coronary heart disease was excluded), the SA group, the UA group, and the AMI group. All the subjects signed the informed consent form for clinical research. This research was approved by the Ethics Committee of Yangzhou University.

### Cardiovascular risk factors

Related risk factors such as gender, weight, age, smoking history, diabetes history, hypertension history, etc. were obtained from all the subjects through questionnaire surveys according to protocols [9].

### Blood biochemical test

Venous blood samples were obtained in the early morning after admission. By using the fully automatic biochemical analyser (OLYMPUS AU2700, JAPAN), related blood biochemical indicators were detected, including cholesterol (TC, mmol/L), triglycerides (TG, mmol/L), high-density lipoprotein cholesterol (HDL-C, mmol/L), low-density cholesterol lipoprotein (LDL-C, mmol/L), lipoprotein(a)[LP(a), mmol/L], uric acid (UA, mmol/L), etc. All tests were performed at the Clinical Laboratory Center of the Affiliated Hospital of Yangzhou University. The laboratory quality control standards were met in this test.

### Measurement of ASI

*Basic principles* Our study was to record the pressure shock wave when blood pressure was measured by oscillometry with an arterial meter. In the process of blood pressure measurement, the inflation pressure in the cuff gradually decreased after reaching the predetermined maximum value, and the arterial blood flow pressure wave detected in the cuff showed regular changes. When the cuff pressure was higher than the systolic pressure, the brachial artery blood flow was completely blocked. The brachial artery above the cuff expanded due to cardiac contraction, so the cuff pressure showed small fluctuations. When the cuff pressure was lower than the systolic pressure, with the gradual opening of the brachial artery below the cuff, the blood flow increased gradually, and the cuff pressure fluctuation amplitude recorded also increased gradually. When the cuff pressure was equal to the average pressure, the pressure fluctuation in the cuff reached the maximum. After that, the cuff pressure decreased gradually, and the pressure fluctuation decreased accordingly. When the cuff pressure was equal to the diastolic pressure, the pressure fluctuation amplitude decreased significantly. After that, as the blood flow was completely unblocked, and the pressure fluctuation amplitude in the cuff tensed to be stable. The ASI value was defined as the width value of the pressure fluctuation diagram corresponding to 80% of the peak height of the maximum pressure wave multiplied by 10, We also designed the basic schematic diagram, as shown in Fig. 1b.

*Measurement method* The subject was required to rest at least 15 min before the measurement. During the measurement, the subject took a seated position with the cuff placed on about 5 cm above the elbow joint of the right upper limb. The maximum inflation pressure of the air cuff (maximum pressure was 300 mmHg) was dependent on the blood pressure of the subjects. When the internal pressure of the cuff reached the maximum inflation pressure, inflation was automatically stopped. Then the cuff automatically deflated at a constant rate. After deflation, ASI, systolic blood pressure (SBP), diastolic blood pressure (DBP), pulse pressure and heart rate could be automatically displayed. During the measurement, the subject kept normal breathing with the whole body relaxed, and avoided moving arms. The measurement was repeated for 3 times to obtain the average value. The time interval between adjacent measurements was more than 5 min. Japanese CardioVision MS-2000 arterial stiffness measuring instrument was used in this experiment.

### Receiver operating curve (ROC) analysis and its significance

Medcalc software was used to draw the ROC. The area under the curve (AUC) was compared and the cut-off value of ASI evaluated, which were used to determine the occurrence and severity of CHD. The severity of CHD was mainly judged by comparing single-vessel lesions with multiple-vessel lesions.

### Statistical analysis

In this study, SPSS 22.0 (Chicago, IL, USA) statistical software package was used for data analysis. For the data conforming to the normal distribution, mean ± standard deviation was used. One-way analysis of variance or independent sample t test was applied to compare the differences between groups. The chi-square test was used in analyzing the proportional data. For the data not conforming to the normal distribution, the median and interquartile range was used. Univariate and multivariate regression analysis was used to evaluate the relationship between the severity of CHD and ASI value. The *P* value was two-sided, and *P* ≤ 0.05 was considered statistically significant.

## Results

A total of 726 patients participated in the study. All patients underwent coronary angiography. According to the results of the examinations, 562 participants were diagnosed with CHD (at least one vessel with vasculopathy degree ≥ 50%), while the other 164 patients were regarded as control group (no one vessel with vasculopathy degree ≥ 50%). Among patients with CHD, there were 340 patients in the single-vessel disease group and 222 patients in the multi-vessel disease group. According to the severity of clinical manifestations, among patients with CHD, there were 164 patients suffered from stable angina, 236 patients unstable angina and 162 patients with acute myocardial infarction.

### The relationship between ASI degree and severity of coronary artery stenosis

As shown in Table 1, according to the number of diseased branches of coronary artery, all the participants were assigned into three groups. Compared with the control group, the participants were elder in single-vessel stenosis group and the multi-vessel stenosis group. In addition, hypertension, FBG, and ASI levels were significantly elevated in the single-vessel stenosis group, and there was no significant difference in other aspects. In the multi-vessel stenosis group, the levels of LDL-C, Lp(a), URIC, FBG, ASI were significantly increased, and there was no significant difference in other aspects. Compared with the single-vessel stenosis group, age, hypertension severity, Lp(a), LDL-C, URIC, and ASI were remarkably increased in the multiple-vessel disease group. After adjusting for baseline data such as male, age, BMI, smoking, hypertension, diabetes, HDL-C, LDL-C, TC, TG, Lp(a), URIC, and FBG, there was still an independent correlation between ASI level and the severity of coronary artery disease, with an odd ratio (OR) of 1.031 (multi-vessel stenosis group vs control group, 95% confidence interval (CI) 1.023–1.040, *P* < 0.05).

**Table 1:** The relationship between ASI degree and severity of coronary artery stenosis

Group	Control	Single	Multiple	*P*
N	164	340	222	
Male (%)	125 (76)	259 (76)	166 (75)	0.919
Age (years)	59.94 ± 11.03	63.46 ± 10.38^#^	66.18 ± 9.92^#^*	< 0.001
BMI (Kg/m^2^)	25.16 ± 3.04	24.99 ± 3.95	24.95 ± 3.28	0.832
Smoking (%)	72(44)	141 (41)	93 (42)	0.871
Hypertension (%)	78(48)	219(64)^#^	169(76)^#^*	< 0.001
Diabetes (%)	19(12)	50(15)	44(20)	0.073
HDL-C (mmol/L)	1.08 ± 0.29	1.04 ± 0.23	1.02 ± 0.53	0.365
LDL-C (mmol/L)	2.14 ± 0.62	2.18 ± 0.74	2.33 ± 0.76^#^*	0.022
TC (mmol/L)	3.78 ± 0.81	3.76 ± 0.96	3.90 ± 0.96	0.186
TG (mmol/L)	2.14 ± 0.62	2.18 ± 0.74	2.33 ± 0.76	0.057
Lp(a)(mmol/L)†	159(113–248)	193(104.25–277.75)	231(133–326)^#^*	0.002
URIC(mmol/L)	364.09 ± 90.89	368.01 ± 85.68	389.62 ± 86.94^#^*	0.005
FBG (mmol/L)†	5.08(4.37–5.93)	5.75(4.93–6.34)^#^	5.71(5.18–6.52)^#^	< 0.001
ASI†	48 (38–57)	80.5(51–131)^#^	134.5(79–210)^#^*	< 0.001

^†^indicated median and inter-quartile range, HDL-C = high density lipoprotein-cholesterol, LDL-C = low density lipoprotein-cholesterol, TC = total cholesterol, TG = triglyceride, Lp(a) = lipoprotein(a), URIC = uric acid, FBG = fasting blood glucose, ASI = Arterial stiffness index. ^#^*P* < 0.05 versus control group, *P < 0.05 versus single group

### The relationship between ASI level and severity of clinical manifestations

According to the severity of clinical manifestations, all the participants were assigned into four groups (Table 2). Compared with the control group, FBG and ASI levels were significantly elevated in the SA group. In the UA group, age, hypertension, URIC, FBG, and ASI etc. were remarkably increased. Age, hypertension levels, TG, FBG, ASI, etc. were dramatically higher in the AMI group. Compared with the SA group, age, h hypertension, URIC, ASI in the UA group were significantly increased. Age, hypertension, TG, and ASI were remarkably elevated in the AMI group. Compared with the UA group, BMI, TG, etc. were significantly increased in the AMI group, but there was no significant difference in ASI. After adjusting for baseline data such as male, age, BMI, smoking, hypertension, diabetes, HDL-C, LDL-C, TC, TG, Lp(a), URIC, and FBG, there was still an independent correlation between ASI level and the severity of clinical manifestations, with odd ratio (OR) of 1.029 (AMI group vs control group, 95% confidence interval (CI) 1.020–1.038, *P* < 0.05).

**Table 2:** The relationship between ASI level and severity of clinical manifestations

Group	Control	SA	UA	AMI	*P*
N	164	164	236	162	
Male (%)	125(76)	122(74)	175(74)	128(79)	0.695
Age (years)	59.94 ± 11.03	60.45 ± 10.47	66.55 ± 10.15^#^*	65.73 ± 9.07^#^*	< 0.001
BMI (Kg/m^2^)	25.16 ± 3.04	24.92 ± 3.76	25.41 ± 4.00	24.40 ± 3.07^&^	0.045
Smoking (%)	72(44)	72(44)	86(36)	76(47)	0.167
Hypertension (%)	78(48)	93(57)	175(74)^#^*	120(74)^#^*	< 0.001
Diabetes (%)	19(12)	23(14)	40(17)	31(19)	0.242
HDL-C (mmol/L)	1.08 ± 0.29	1.07 ± 0.25	1.00 ± 0.22	1.05 ± 0.60	0.148
LDL-C (mmol/L)	2.14 ± 0.62	2.32 ± 0.81	2.16 ± 0.73	2.27 ± 0.72	0.057
TC (mmol/L)	3.78 ± 0.81	3.92 ± 0.93	3.71 ± 0.97	3.86 ± 0.96	0.133
TG (mmol/L)	1.51 ± 0.70	1.62 ± 0.77	1.64 ± 0.79	1.81 ± 0.87^#^*^&^	0.007
Lp(a) (mmol/L)	159(113–248)	199(105.5–327.5)	193(105–291.5)	235(140.25–316.5)	0.088
URIC (mmol/L)	364.09 ± 90.89	366.81 ± 94.69	387.18 ± 80.63^#^*	370.90 ± 85.80	0.033
FBG (mmol/L)	5.08(4.37–5.93)	5.79(4.96–6.39)^#^	5.83(5.01–6.39)^#^	5.49(5.11–6.22)^#^	< 0.001
ASI	48(38–57)	57(43–79)^#^	133(90–170.50)^#^*	120(67–170.75) ^#^*	< 0.001

^†^indicated median and inter-quartile range, ^#^*P* < 0.05 versus control group, *P < 0.05 versus SA group, ^&^*P* < 0.05 versus UA group

### Using ASI cut-off value in the diagnosing CHD and evaluating its severity

**Fig. 2:**
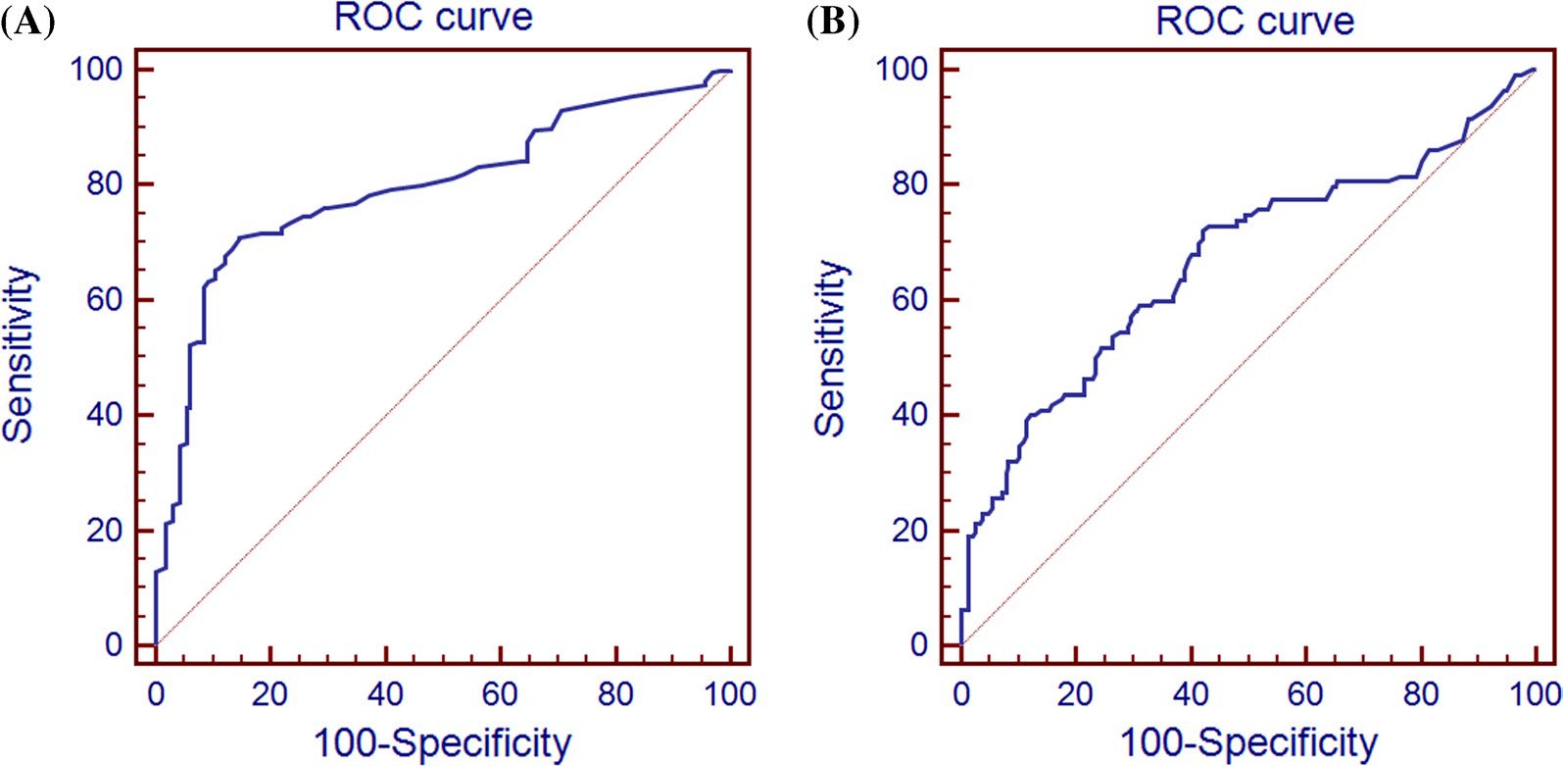
Using ASI cut-off value in the diagnosing CHD and evaluating its severity. **a** ROC analysis results for ASI in assessing the presence of CHD; **b** ROC analysis results for ASI in assessing the severity of CHD

ROC analysis was used to determine the role of ASI in diagnosing CHD and assessing its severity (Fig. 2). The areas under the curve (AUC) in Fig. 2a and b was 0.795 and 0.668, respectively. The sensitivity and specificity of ASI were 71.0% and 85.4% in diagnosing CHD. The sensitivity and specificity of ASI were 72.1% and 57.9% in predicting the severity of CHD.

## Discussion

Coronary arteriosclerosis is one of the important causes of CHD [11], and blood vessel elasticity is closely related to coronary arteriosclerosis. Previous studies have demonstrated that ASI could indicate the compliance of the systemic vascular system, especially large blood vessels [8, 12, 13]. Thus, it was believed that by detecting ASI, the occurrence and severity of ischemic cardiovascular disease, especially coronary heart disease could be evaluated. Our study confirmed that the ASI level was significantly higher in the patients with CHD than that of the normal people. The ASI level in the patients with multi-vessel stenosis was remarkably higher compared with single-vessel stenosis. With the aggravation of clinical manifestations, the ASI of patients in the UA group and AMI group was significantly increased compared with the normal and SA group. To a certain extent, these findings confirmed that ASI was closely related to CHD and its severity. The reason why ASI could have such an effect is a question worthy of further investigation.

As is known to all, vascular endothelial cells can synthesize and release a variety of vasoactive substances, which play a vital role in maintaining vascular functions, such as vasodilation, inhibition of platelet adhesion, and the proliferation of vascular smooth muscle cell [14, 15]. Previous studies have presented that some risk factors, such as age, blood sugar imbalance, hypertension, blood lipid level imbalance, etc. could cause vascular endothelial damage, which in turn contributed to an imbalance of vasoactive factors secreted by endothelial cells. These could result in the structural changes of the vascular wall, and ultimately decreased vascular compliance [16]. Our research showed that there were significant differences in basic data such as age, hypertension, and blood sugar levels in the patients with CHD compared with normal people. There was also obvious difference in blood lipid levels in some subgroups. With the increase in the number of vessels diseased and the aggravation of clinical manifestations, the difference was also increased. These factors with significant difference were risk factors for vascular endothelial damage. Arterial endothelial injury leads to changes in arterial wall structure and diastolic and systolic function, which is the main reason for the decrease in arterial elasticity [17]. Therefore, our results further confirmed this view.

Reduced vascular compliance resulted from endothelial dysfunction generally occurs earlier than atherosclerotic plaque formation, which is the earliest manifestation of vasculopathy [18, 19]. The essence of CHD is the result of coronary artery disease [20]. Higher ASI level indicated that the arterial elasticity is worse. Presented by logistic regression analysis, ASI was not only related with the number of vessels diseased, but also with the clinical classification of CHD. Loss of arterial elasticity is an independent risk factor for CHD, this is consistent with the results in the research by Nigam et al. [21]. It was believed that endothelial function damage is the key link between them. In summary, with the increase of age and influence of some vascular-related risk factors, vascular endothelium is injured, resulting in decreased vascular compliance, this further contributes to vascular sclerosis, and thereby promoting the occurrence and development of CHD.

Our study was the first research using ROC analysis to determine the diagnostic value of ASI for CHD. The results of ROC analysis showed that ASI could not only serve a part in the predictive diagnosis of CHD, but also played a pivotal role in assessing the severity of CHD. Higher ASI value indicated that CHD was more serious. There are also some limitations in our research. First of all, our research time was relatively short and the number of cases was relatively small. Secondly, we have not performed vascular endothelial cell function test, which may require further validation in future animal experiments. Finally, for some patients with microvascular disease (without actual clinical significance), their test results might have some impact on our research, however, we believed that these impacts were limited.

## Conclusions

In short, as one of the indicators reflecting vascular compliance, ASI closely related to the severity of CHD. Our research results demonstrated that ASI could provide a new basis for predicting and diagnosing CHD, which also could provides a new idea for the prevention and treatment of CHD in the future.

## Data Availability

The datasets used and/or analysed during the current study are available from the corresponding author on reasonable request.

## Abbreviations

ASI: Arterial stiffness index
CHD: Coronary heart disease
SA: Stable angina
UA: Unstable angina
AMI: Acute myocardial infarction
ROC: Receiver operating curve
PWV: Pulse wave velocity
SBP: Systolic blood pressure
DBP: Diastolic blood pressure
AUC: Area under the curve
FBG: Fasting blood glucose
Lp(a): Lipoprotein(a)
TC: Total cholesterol
TG: Triglyceride
LDL-C: Low-density lipoprotein-cholesterol
HDL-C: High-density lipoprotein-cholesterol
URIC: Uric acid
